# Radiation effect on stagnation point flow of Casson nanofluid past a stretching plate/cylinder

**DOI:** 10.1038/s41598-024-51963-2

**Published:** 2024-01-16

**Authors:** U. S. Mahabaleshwar, T. Maranna, Manoranjan Mishra, M. Hatami, Bengt Sunden

**Affiliations:** 1 Department of Studies in Mathematics, Davangere University, Shivagangothri, Davangere, Karnataka 577007 India; 2 Department of Mathematics, Indian Institute of Technology Ropar, Rupnagar, Punjab 140001 India; 3 Department of Mechanical Engineering, Esfarayen University of Technology, Esfarayen, Iran; 4 Lund University, 22100 Lund, Sweden

**Keywords:** Materials science, Mathematics and computing, Physics

## Abstract

The exclusive behaviour of nanofluid has been actively emphasized due to the determination of improved thermal efficiency. Hence, the aim of this study is to highlight the laminar boundary layer axisymmetric stagnation point flow of Casson nanofluid past a stretching plate/cylinder under the influence of thermal radiation and suction/injection. Nanofluid comprises water and *Fe*_*3*_*O*_*4*_ as nanoparticles. In this article, a novel casson nanofluid model has been developed and studied on stretchable flat plate or circular cylinder. Adequate rational assumptions (velocity components) are employed for the transformation of the governing partial-differential equations into a group of non-dimensional ordinary-differential formulas, which are then solved analytically. The momentum and energy equations are solved through the complementary error function method and scaling quantities. Using various figures, the effects of essential factors on the nanofluid flow, heat transportation, and Nusselt number, are determined and explored. From obtained results, it is observed that the velocity field diminishes owing to magnification in stretching parameter $B$ and Casson fluid parameter $\Lambda$. The temperature field increases by amplifying radiation $N_{r}$, and solid volume fraction parameter $\phi$. The research is applicable to developing procedures for electric-conductive nanomaterials, which have potential applications in aircraft, smart coating transport phenomena, industry, engineering, and other sectors.

## Introduction

The fluid movement near a solid surface’s stagnation area is referred to as stagnation point flow. The stagnation flows occur in a variety of application fields, such as submarines, aircraft, and flows over the tips of rockets. The concern of stagnation point flow has been expanded in a variety of approaches, including boundary layer. Historically, Hiemenz^1^ was a pioneering researcher who considered the issue of two-dimensional stagnation point flow towards a flat surface and found that the Navier–Stokes formulas may accurately examine this problem. Homann^2^ expanded this issue to include the scenario of axisymmetric stagnation-point circulation. After that, Eckert^3^ strengthened the suggested formulation by inserting both energy and momentum. Very recently, Norzawary^4^ investigated the difficulties of stagnation point flow in carbon nanotubes through a stretching/shrinking sheet and the effects of suction/ injection on it. Shah^5^ tackles the problem of boundary layer flow and heat transmission analysis of coupled stress fluid stagnation point flows through a continuously stretched surface. Soid^6^ employs a numerical model to deliberate an axisymmetric stagnation point flow past a stretching/shrinking sheet through second order slip as well as a thermal jump. Another popular topic of current research activity is stagnation-point flows across extending/contracting bodies. For instance, Merkin^7^ addresses the movement of the boundary layer and the transfer of heat through a cylinder that is exponentially expanding and contracting. Turkyilmazoglu^8^ has looked at how exactly the solution arises from stagnation-point flows through stretched flat plates or circular pipes.

The casson fluid constitutes one of the most prominent non-Newtonian rheological concepts, and it is a plastic fluid with significant yield stress and shear-subordinate characteristics. The Casson fluid flow happens whenever the shear tension exceeds the yield stress. The Casson model, which is developed for fluids with bar-like texture materials along it, is commonly used to simulate plasma flow and other real-world scenarios, including manipulating liquid chocolate and comparable meals in the present era. The presence of a stretched barrier expands under a variety of conditions, including the flow induced by the removal of polymers, the designing of copper cables, the persistent extension of synthetic films and reconstituted threads, the movement of hot glass fibers, the expulsion of metal, and material twisting. Researchers are more interested in conducting theoretical and practical studies on the flow properties of such complicated fluids, as non-Newtonian fluids are used in industrial applications more frequently every day. The boundary layer expressions among motion generated viz stretched plastic sheets in the polymer industry were analytically solved by Sakiadis^9,10^ and Crane^11^. Very recently, Mahanta^12^ demonstrated the effect of magnetohydrodynamic stagnation point flow on non-Newtonian liquid past a strengthening sheet. Mahabaleshwar^13^ has analytically presented radiative magnetohydrodynamic axisymmetric flow of Casson fluid over a stretching/shrinking sheet in porous medium. Rehman^14^ investigates concurrent heat and mass transfer properties of the flow of Casson fluid approaching aligned stretched flat and cylindrical surfaces. Devdas^15^ has developed computational dual remedies for stagnation point flow and molten thermal expansion of non-Newtonian liquid due to a stretched surface.

On the other hand, in order to control conventional heat transfer fluids like ethylene glycol, oil, and water, nanoparticles with an average size of less than 100 nm are suspended in the fluids. They are distinguished by the enrichment of typically used fluids, such as $H_{2} O$, toluol, ethylene alcohol, and fuel, along with nanomaterials of different kinds, like metals, oxides, carbides, and carbon. *TiO*_*2*_*, CuO, Al*_*2*_*O*_*3*_*, and ZnO* are examples of common nanofluids, along with water. Nanofluids are currently intended to have huge implications in pharmaceuticals, biomedicines, deterrence, nuclear power generation, and, more specifically, any heat dissipation associated with industrial uses. Choi^16^, the person who introduced the term “nanofluid,” has conducted substantial studies on non-Newtonian flow developments and applications as well as optimizing the nanofluids heat conductivity. Yang^17^ scrutinized the heat transfer in casson nanofluid flowing at a stagnation point flow across a shrinking sheet with viscous dissipation. Mahabaleshwar^18,19^ highlights the erratic stagnation point flow of a hybrid nanofluid through a stretching/shrinking sheet inserted in a permeable material with mass transpiration and chemical processes. Poornima^20^ studied steady two dimensional, immiscible boundary layer stagnation point flow of regular and hybrid *CuO* + *MgO* nanoparticles across a stretching/shrinking cylinder. As a result of this, several more analytical and numerical research on the flow of nanoparticles has been widely published, such as in references^21–26^.

The effect of radiation on the flow of a nanofluid has become very important industrially. At very high operating temperature, the effects of radiation can be very significant. In engineering areas many processes occur at high temperature and the knowledge of radiation heat transfer becomes very important for designing of reliable equipment’s, nuclear plants, turbines of gas and several devices of propulsion or aircraft, missiles, satellites and space vehicles. Nayak^27^ presents the impacts of slip velocity, radiation, and magnetohydrodynamic consequences when they are taken into account at the stagnation point flow over the stretching sheet. Mahabaleshwar^28^ also describes an analytical method for quantifying the impact of incompressible, casson nanofluid fluid through stretching/shrinking sheet in the existence of thermal radiant mass transmission characteristics. Kirankumar^29^ considers the unstable magnetohydrodynamic boundary layer stagnation point flow of nanofluid across a non-linear elongated sheet along a permeable media with a fluctuating sheet. Daniel^30^ deliberates on the impact of radiation and MHD on a flow toward an extended surface in a two-dimensional stagnation point. Kaneez^31^ explored the thermal efficiency of Non-Newtonian fluid by submerging trihybrid nanoparticles *Fe*_*3*_*O*_*4*_*–Al*_*2*_*O*_*3*_*–TiO*_*2*_ uniformly. Aside from these, there have been a few recent studies^32–38^ on the impact of thermal radiation on the various nanoparticles flow past a stretching/shrinking surface or different geometries. Recently, Maranna^39^ presented an effect of radiation and mass suction/injection on two dimensional laminar flow of ternary nanoparticles due to porous stretching/shrinking sheet.

The above-mentioned literature survey reveals that no research work exists on axisymmetric stagnation point flow of Casson nanofluid over a stretching flat plates/circular cylinder. Also, there does not exist any solution to such a type of problem analytically in the complementary error function form. Hence, the novelty of this paper is to examine the axisymmetric stagnation point flow of Casson nanofluid over a stretching flat plates/circular cylinder. For both cartesian/circular cylindrical coordinate systems, the regulating PDEs are turned into a system of ODEs form via a velocity component and then solved analytically. For both momentum and energy fields, precise solutions are desired. The momentum and energy equations have precise solutions as a result of the established analytical solutions, which is important. The effects of the stretching parameter, Casson parameter, mass suction/injection, solid volume fraction, and radiation will also be analyzed through graphs.

The following are the innovative aspects of the planned investigation:

- The importance of heat radiation effects on axisymmetric stagnation point flow of non-Newtonian liquid through a stretching flat sheet/circular cylinder.
- The exact solution also determines whether to injection/suction into the walls and when stretching is not present, these solutions tend to the well-known particular limitations.
- Consider Fe_3_O_4_ nanoparticles suspended in base fluid as water.
- The collective consequences of the stretching, permeability factors, solid volume fraction, and thermal radiation are ameliorated in this simulation.
- The use of nanoparticles in microprocessors, fuel cells, pharmacological procedures, hybrid-powered motors, ignition stimulating, the control of vehicle temperature, domestic refrigerators, coolers, heat exchangers, atomic power plant, grinding, machining, satellite communications, defensive performance, ships, and boiler flue gas thermal reduction have all been the subject of ongoing studies.

## Mathematical modelling and solution

**Figure 1:**
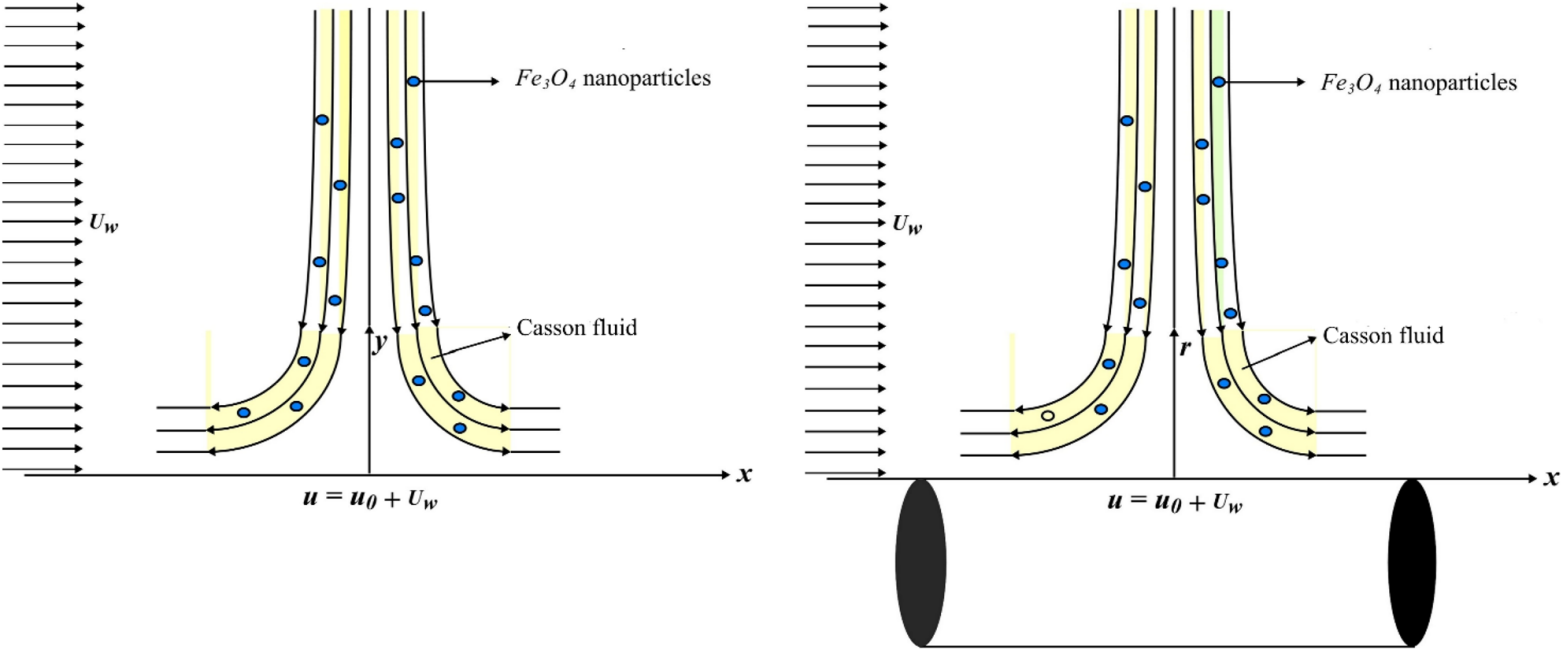
Flow model drawing.

Assume two dimensional laminar axisymmetric stagnation point flow of non-Newtonian fluid across a flat stretching sheet /a circular cylinder that can be stretched. Allow an encroaching flow on a sheet to collide with an approaching flow with axial velocity $u\, = \,U_{w} \left( x \right)\, = \,\alpha x$. The surfaces should extend with an assumed velocity $u\, = \,U_{w} \left( x \right) + u_{0}$, in which $u_{0}$ is fixed in both flow configurations as shown in Fig. 1. The two dimensional momentum field involves axial $x$ factor as $u$ and radial $y$ factor as $v$. In addition, using both radial and axial dimensions, p indicates the pressure exerted on the system. $T$ is considered to be fluid temperature, uniform wall temperature is signified as $T_{w}$ and $T_{\infty }$ is the ambient temperature, respectively.

### Inferences and model expressions

The followings are the aspects and problems that concern the mobility system:

- Two dimensional laminar boundary layer axisymmetric stagnation point flow.
- Non-Newtonian casson nanofluid.
- Flat stretching sheet.
- Circular stretching cylinder.
- Variable thermal radiation.

### Rheological expression for Casson fluid

Assuming rheological equation of non-Newtonian Casson fluid as^40,41^:

1 $$\tau_{i,j} \, = \,\left\{ \begin{gathered} \left( {\mu_{B} + \frac{{p_{y} }}{{\sqrt {2\pi } }}} \right)2e_{ij} ,\,\,\,\,\,\,\,\pi > \pi_{c} \hfill \\ \left( {\mu_{B} + \frac{{p_{y} }}{{\sqrt {2\pi } }}} \right)2e_{ij} ,\,\,\,\,\,\,\,\pi < \pi_{c} \hfill \\ \end{gathered} \right.,$$

In the above equation, $\mu_{B}$ is the dynamic viscosity of the biviscous Bingham fluid, fluid yield stress is $p_{y}$, $\pi$ is the result of multiplying the deformation rate components by itself, notably, $\pi \, = \,e_{ij} e_{ij}$, $e_{ij}$ is the $\left( {i,j} \right)$th components of the deformation rate, and $\pi_{c}$ is the critical values of the $\pi$.

### Geometrical configuration

### Formal equalities

#### Spherical coordinates

Under the foregoing assumptions, the governing boundary layer equations can be described^8,42^ as

2 $$\frac{\partial u}{{\partial x}} + \frac{\partial v}{{\partial y}} = 0,$$

3 $$u\frac{\partial u}{{\partial x}} + v\frac{\partial u}{{\partial y}}\, = \, - \frac{1}{{\rho_{nf} }}\frac{\partial p}{{\partial x}} + \left( {1 + \frac{1}{\Lambda }} \right)\nu_{nf} \left( {\frac{{\partial^{2} u}}{{\partial x^{2} }} + \frac{{\partial^{2} u}}{{\partial y^{2} }}} \right),$$

4 $$u\frac{\partial v}{{\partial x}} + v\frac{\partial v}{{\partial y}}\, = \, - \frac{1}{{\rho_{nf} }}\frac{\partial p}{{\partial y}} + \left( {1 + \frac{1}{\Lambda }} \right)\nu_{nf} \left( {\frac{{\partial^{2} v}}{{\partial x^{2} }} + \frac{{\partial^{2} v}}{{\partial y^{2} }}} \right),$$

5 $$u\frac{\partial T}{{\partial x}} + v\frac{\partial T}{{\partial y}}\, = \,\chi \left( {\frac{{\partial^{2} T}}{{\partial x^{2} }} + \frac{{\partial^{2} T}}{{\partial y^{2} }}} \right) - \frac{{\partial q_{r} }}{\partial y},$$

#### Cylindrical coordinates

Under certain assumptions, the fundamental equations in cylindrical coordinates $\left( {r,x} \right)$ are as follows^7,8,53,54^:

6 $$\frac{1}{r}\frac{{\partial \left( {ru} \right)}}{\partial r} + \frac{\partial u}{{\partial x}}\, = \,0,$$

7 $$u\frac{\partial u}{{\partial x}} + v\frac{\partial u}{{\partial r}} = - \frac{1}{{\rho_{nf} }}\frac{\partial p}{{\partial x}}\left( {1 + \frac{1}{\Lambda }} \right)\nu_{nf} \left( {\frac{{\partial^{2} u}}{{\partial r^{2} }} + \frac{1}{r}\frac{\partial u}{{\partial r}} + \frac{{\partial^{2} u}}{{\partial x^{2} }}} \right),$$

8 $$u\frac{\partial v}{{\partial x}} + v\frac{\partial v}{{\partial r}}\, = \, - \frac{1}{{\rho_{nf} }}\frac{\partial p}{{\partial r}} + \left( {1 + \frac{1}{\Lambda }} \right)\nu_{nf} \left( {\frac{{\partial^{2} v}}{{\partial r^{2} }} + \frac{1}{r}\frac{\partial v}{{\partial r}} + \frac{{\partial^{2} v}}{{\partial x^{2} }}} \right),$$

9 $$u\frac{\partial T}{{\partial x}} + v\frac{\partial T}{{\partial r}} = \chi \left( {\frac{{\partial^{2} T}}{{\partial r^{2} }} + \frac{1}{r}\frac{\partial T}{{\partial r}} + \frac{{\partial^{2} T}}{{\partial x^{2} }}} \right) - \frac{1}{{\left( {\rho C_{p} } \right)_{nf} }}\frac{1}{r}\frac{\partial }{\partial r}\left( {rq_{r} } \right),$$

It should be highlighted that the hydraulic and thermal characteristics are supposed to be constant and independent of any external factors.

Given the aforementioned reasoning assumptions, in both configurations, the axial velocities are being addressed in the following manner^8^:

10 $$\begin{gathered} u = U_{w} \left( x \right) + U\left( y \right), \hfill \\ u = U_{w} \left( x \right) + U\left( r \right). \hfill \\ \end{gathered}$$

And the solution for radial velocity can be described as^8^:

11 $$\begin{gathered} v\, = \, - \alpha y - v_{0} , \hfill \\ v\, = \, - \frac{\alpha r}{2}. \hfill \\ \end{gathered}$$

where velocity constituents in $\left( {x,y} \right)$ are $\left( {u,v} \right)$ correspondingly, $\left( {x,r} \right)$ are axial and radial constituents of $\left( {u,v} \right)$. According to both axial and radial dimensions, $p$ is symbolizes the pressure that is exerting itself on the system. $\rho_{nf}$ is the nanofluid density, $\nu_{nf}$ kinematic viscosity of the nanofluid is denoted as $\nu_{nf}$, $\Lambda \left( { = \frac{{\mu_{B} \sqrt {2\pi_{c} } }}{{p_{y} }}} \right)$ is the Casson parameter, and $\chi \, = \,\frac{{\kappa_{nf} }}{{\left( {\rho C_{p} } \right)_{nf} }}$ is the thermal diffusivity of the nanofluid, whereas $\kappa_{nf}$ is the thermal conductivity of the nanofluid and $\left( {\rho C_{p} } \right)_{nf}$ is heat capacity of nanofluid. It is noted that the stretched flow over a smooth surface across $v_{0}$ in (11) may have a cumulative rate of suction/injection. At velocities less than $- \frac{\alpha R}{2}$, only suction can be imparted to the pipe surface with $R$ is the pipe radius. Furthermore, the stagnation-point flow occurs beneath the fluid flows provided by Systems (6–9) and (11).

### Boundary constraints

The related boundary constraints^8^ are

12 $$\begin{gathered} U\left( y \right)\, = \,u_{0} ,\,\,\,\,\,\,\,\,U\left( {y \to \infty } \right)\, = \,\frac{{\delta _{1} \nu _{{nf}}^{2} }}{\alpha }, \hfill \\ U\left( {r\, = \,1} \right)\, = \,u_{0} ,\,\,\,\,U\left( {r \to \infty } \right)\, = \,\frac{{\delta _{1} \nu _{{nf}}^{2} }}{\alpha }, \hfill \\ T\left( {y = 0} \right)\, = T_{w} ,\,\,\,\,\,\,\,\,T\left( {y\, \to \infty } \right)\, = \,T_{\infty } , \hfill \\ {\text{and}} \hfill \\ T\left( {r = R} \right)\, = \,T_{w} ,\,\,\,\,\,T\left( {r\, \to \infty } \right)\, = \,T_{\infty } . \hfill \\ \end{gathered}$$

### Similarity transformation for energy equation

The desired similarity quantity^37^ is

13 $$\theta \left( y \right)\, = \,\frac{{T - T_{\infty } }}{{T_{w} - T_{\infty } }},$$

whenever the flat-plate scenario and the Navier’s-stokes equations in (2–9) are taken into account, the pressure solution is obtained^8^.

14 $$\frac{p}{\rho }\, = \, - \frac{{\alpha^{2} }}{2}\left( {x^{2} + y^{2} } \right) - \alpha v_{0} y - \delta_{1} \nu_{nf}^{2} x + p_{0} ,$$

where $p_{0}$ and $\delta_{1}$ are being constants.

### Analysis of Rosseland approximations

While this is happening, the Rosseland approach for heat radiation is provided^43–46^ as

15 $$q_{r} \, = \, - \frac{{4\sigma^{*} }}{{3k^{*} }}\frac{{\partial T^{4} }}{\partial y},$$

where $\sigma^{*}$ is the Stefan-Boltzmann constant and $k^{*}$ is known as mean absorption coefficient. Suppose that the temperature fluctuations inside the flow are negligibly infinitesimal, allowing $T^{4}$ to be represented as a temperature-dependent linear function $T^{4} \,\, \cong \,4T_{\infty }^{3} T - 3T_{\infty }^{4}$, we have

16 $$\frac{{\partial q_{r} }}{\partial y}\, = \, - \frac{{16\sigma^{*} T_{\infty }^{3} }}{{3k^{*} }}\frac{{\partial^{2} T}}{{\partial y^{2} }},$$

Consequently, after simplifying the Eqns. (10) and (11), axial velocity profile $U\left( y \right)$ ought to be deduced from the boundary layer problem.

17 $$\left( {1 + \frac{1}{\Lambda }} \right)\nu_{f} \Gamma \frac{{d^{2} U}}{{dy^{2} }} + \left( {\alpha y + v_{0} } \right)\frac{dU}{{dy}} - \alpha U\left( y \right)\, = \, - \delta_{1} \Gamma^{2} \nu_{f}^{2} ,$$

18 $$\left( {1 + \frac{1}{\Lambda }} \right)\nu_{f} \Gamma \frac{{d^{2} U}}{{dr^{2} }} + \left( {\frac{\alpha r}{2} + \frac{1}{r}} \right)\frac{dU}{{dr}} - \alpha U\left( r \right)\, = \, - \delta_{1} \Gamma^{2} \nu_{f}^{2} ,$$

where $\Gamma \, = \,\frac{{\mu_{nf} /\mu_{f} }}{{\rho_{nf} /\rho_{f} }}$ is the dummy variable, whereas $\mu_{nf}$ is the dynamic viscosity of nanofluid. The condition $\,\,U\left( {y \to \infty } \right)\, = \,\frac{{\delta_{1} \Gamma^{2} \nu_{f}^{2} }}{\alpha }$ in Eq. (12) is due to the velocity-dependent asymptotic decay characteristic.

The non-dimensional variables should be established and stated^8^ as follows:

19 $$U = \overline{U} \,\frac{{v_{f} }}{L},\,\,\,v\, = \,\frac{{v_{f} }}{L}\overline{v} ,\,\,\,\,x\, = \,\overline{x} L,\,\,\,y\, = \,\overline{y} L,\,\,\,p\, = \,\overline{p} \frac{{\nu_{f}^{2} \rho_{f} }}{{L^{2} }},\,r\, = \,\overline{r} L.$$

where $L$ is the reference length scale, incorporating these scalings and eliminating the bars, Eqns. (6) and (7) becomes

20 $$\left( {1 + \frac{1}{\Lambda }} \right)\Gamma \frac{{d^{2} U}}{{dy^{2} }} + \left( {By + v_{0} } \right)\frac{dU}{{dy}} - BU\left( y \right)\, = \, - \delta ,$$

21 $$\left( {1 + \frac{1}{\Lambda }} \right)\Gamma \frac{{\partial^{2} U}}{{\partial r^{2} }} + \left( {\frac{Br}{2} + \frac{1}{r}} \right)\frac{\partial U}{{\partial r}} - BU\left( r \right)\, = \, - \delta ,$$

Corresponding to transformed boundary conditions are

22 $$\begin{gathered} U\left( {y\, = \,0} \right)\, = \,u_{0} ,\,\,\,\,\,\,\,\,U\left( {y \to \infty } \right)\, = \,\frac{\delta }{B}, \hfill \\ U\left( {r = 1} \right)\, = \,u_{0} ,\,\,\,\,\,\,\,\,\,U\left( {r \to \infty } \right)\, = \,\frac{\delta }{B}. \hfill \\ \end{gathered}$$

where $B\, = \,\frac{{\alpha L^{2} }}{{\nu_{f} }}$ is the stretching parameter, permeability is designated as $s\, = \,\frac{{v_{0} L}}{{\nu_{f} }}$, and $\delta \, = \,\delta_{1} \Gamma^{2} L^{3}$ is the pressure parameter.

## Analytical solution for the momentum equations

In light of the complementary error function ${\text{Erfc}}\,{ = }\,{1} - {\text{Erf}}$, the axial velocity from Eq. (20) is determined in relation to stretching flow across an obstacle.

23 $$U\left( y \right)\, = \,\frac{{e^{{ - \frac{{2y^{2} \Lambda }}{{2\Gamma \left( {1 + \Lambda } \right)}} - \frac{sy\Lambda }{{\Gamma \left( {1 + \Lambda } \right)}}}} \left\{ \begin{gathered} - 2\sqrt B \left( {Bu_{0} + \left( {\delta e^{{\frac{sy\Lambda }{{\Gamma \left( {1 + \Lambda } \right)}} + \frac{{By^{2} \Lambda }}{{2\Gamma \left( {1 + \Lambda } \right)}}}} - \delta } \right)} \right) + e^{{\frac{{s^{2} \Lambda }}{{2B\Gamma \left( {1 + \Lambda } \right)}}}} \frac{{\sqrt {\frac{2\pi \Lambda }{{\left( {1 + \Lambda } \right)}}} }}{\sqrt \Gamma }\left( {s\delta Erfc\left( {\frac{{s\sqrt {\frac{\Lambda }{{\left( {1 + \Lambda } \right)}}} }}{{\sqrt {2B\Gamma } }}} \right)} \right) + \hfill \\ \left( {s + By} \right)\left( {\frac{{\left( {u_{0} B - \delta } \right)}}{\sqrt B }} \right)Erfc\left( {\frac{{\left( {s + By} \right)\sqrt {\frac{\Lambda }{{\left( {1 + \Lambda } \right)}}} }}{{\sqrt {2B\Gamma } }}} \right) \hfill \\ \end{gathered} \right\}}}{{\left( { - 2B^{\frac{3}{2}} + \frac{{\sqrt {\frac{2\pi \Lambda }{{\left( {1 + \Lambda } \right)}}} }}{\sqrt \Gamma }Bse^{{\frac{{s^{2} \Lambda }}{{2B\Gamma \left( {1 + \Lambda } \right)}}}} Erfc\left( {\frac{{s\sqrt {\frac{\Lambda }{{\left( {1 + \Lambda } \right)}}} }}{{\sqrt {2B\Gamma } }}} \right)} \right)}}$$

which is analytical solution for momentum expression in spherical form. Now Eq. (23) can be reduced to following for keeping $u_{0} \, = \,s\, = \,0$.

The radial velocity as well as pressure in dimensionless representations, are integrated into the solutions in (23) and (24).

24 $$v\, = \, - By - s,$$

25 $$p\, = \, - \frac{B}{2}\left( {x^{2} + y^{2} } \right) - Bsy - \delta x + \overline{{p_{0} }} ,$$

The results in (23) and (24) are similar in that they disappear at the limit $B\, \to \,\infty$.

## Analytical solution for the energy equation

After employing Eq. (13) into Eqs. (5) and (9), yields

26 $$\left( {A_{1} + N_{r} } \right)\frac{{\partial^{2} \theta }}{{\partial y^{2} }} + \Pr A_{2} \left( {By + s} \right)\frac{\partial \theta }{{\partial y}}\, = \,0,$$

27 $$\left( {A_{1} + N_{r} } \right)\frac{{d^{2} \theta }}{{dr^{2} }} + \left( {\frac{{\Pr BA_{2} r}}{2} + \frac{{A_{1} }}{r}} \right)\frac{d\theta }{{dr}}\, = \,0,$$

with transformed temperature boundary conditions are

28 $$\begin{gathered} \theta \left( {y\, = \,0} \right)\, = \,1,\,\,\,\,\,\,\,\,\,\,\theta \left( {y\, \to \,\infty } \right)\, = \,0, \hfill \\ {\text{and}} \hfill \\ \theta \left( {r\, = \,1} \right)\, = \,1,\,\,\,\,\,\,\,\,\theta \left( {r \to \infty } \right)\, = \,0. \hfill \\ \end{gathered}$$

The following are some of the factors that appear in Eqs. (26) and (27) are:

1. $A_{1} \, = \,\frac{{\kappa_{nf} }}{{\kappa_{f} }},\,\,A_{2} \, = \,\frac{{\left( {\rho C_{p} } \right)_{nf} }}{{\left( {\rho C_{p} } \right)_{f} }}$ are the dummy variables.
2. $\Pr \, = \,\frac{{\mu_{f} C_{p} }}{{\kappa_{f} }}$ is the Prandtl number.
3. $N_{r} \, = \,\frac{{16\sigma^{*} T_{\infty }^{3} }}{{3k^{*} \kappa_{f} }}$ is the radiation parameter.

Equations (26) and (27) differentiating twice, we have obtained following results

29 $$\theta \left( y \right)\, = \,\frac{{Erfc\left( {\frac{{\Pr \sqrt {A_{2} } \left( {By + s} \right)}}{{\sqrt {2B\Pr \left( {A_{1} + N_{r} } \right)} }}} \right)}}{{Erfc\left( {\frac{{\sqrt {\frac{{\Pr A_{2} }}{{B\left( {A_{1} + N_{r} } \right)}}} }}{\sqrt 2 }} \right)}},$$

and

30 $$\theta \left( r \right)\, = \,\frac{{Ei\left( { - \frac{{B\Pr A_{2} r^{2} }}{{4\left( {A_{1} + N_{r} } \right)}}} \right)}}{{Ei\left( {\frac{{ - \frac{{B\Pr A_{2} }}{{\left( {A_{1} + N_{r} } \right)}}}}{4}} \right)}},$$

For the flat stretching sheet, the heat transfer rate from the body surface can then be quantified using normalized Nusselt numbers provided by Fourier's heat equation.

31 $$- \left( {\frac{\partial \theta }{{\partial y}}} \right)_{y\, = \,0} = \,\frac{{e^{{\frac{{\frac{{\Pr A_{2} s^{2} }}{{\left( {A_{1} + R} \right)}}}}{2B}}} \sqrt {\frac{2}{\pi }} \sqrt {\frac{{B\Pr A_{2} }}{{\left( {A_{1} + R} \right)}}} }}{{Erfc\left( {\frac{{\sqrt {\frac{{\Pr A_{2} }}{{B\left( {A_{1} + R} \right)}}} }}{\sqrt 2 }} \right)}},$$

and for the circular cylinder

32 $$- \left( {\frac{\partial \theta }{{\partial r}}} \right)_{r\, = \,1} \, = \, - \frac{{2e^{{ - \frac{{B\Pr A_{2} }}{{\left( {A_{1} + R} \right)}}}} }}{{Ei\left( {\frac{{ - \frac{{B\Pr A_{2} }}{{\left( {A_{1} + N_{r} } \right)}}}}{4}} \right)}}.$$

The present section briefly discussed about precise solution for the energy equation, in the next section we will discuss about outcomes of the present problem.

## Results and discussion

To investigate the impact of non-dimensional regulating factors on Casson nanofluid flow across an extending flat plate or circular cylinder, analytical calculations are specified. In this article, the shapes of stretching flat plates and pipes will be explored in light of the derived velocity and energy solutions. *Fe*_*3*_*O*_*4*_ is thought to be a nanofluid in the current problem, with water acting as a base liquid. Prandtl number $\Pr$ is considered to have a value of 6.2. The impact of the stretching parameter $B$, wall suction/injection parameter $s$, Casson fluid parameter $\Lambda$, radiation $N_{r}$, and solid volume fraction parameter $\phi$ on the momentum, temperature, and Nusselt number profiles are depicted in the plots. To obtain the results in terms of figures the value of parameter ranges between $0.1 \le B \le 10,\;\;\, - 0.25 \le s \le 1,\;\;\,0.1 \le \Lambda \le 1,\,\;\;0.01 \le \phi \le 0.3$,and $1 \le N_{r} \le 3$. The thermophysical properties of nanofluid are mentioned in Tables 1 and 2.

**Table 1:** Mathematical expression for empirical correlation of nanofluid^47–49^.

Thermo-physical properties	Nanofluid ($Fe_{3} O_{4}$)
Density	$\rho_{nf} \, = \,\left( {\left( {1 - \phi } \right) + \phi \left( {\frac{{\rho_{s} }}{{\rho_{f} }}} \right)} \right)\rho_{f}$
Viscosity	$\mu_{nf} \, = \,\frac{{\mu_{f} }}{{\left( {1 - \phi } \right)^{2.5} }}$
Heat capacitance	$\left( {\rho C_{p} } \right)_{nf} \, = \,\left( {\rho C_{p} } \right)_{f} \left( {\left( {1 - \phi } \right) + \phi \left( {\frac{{\left( {\rho C_{p} } \right)_{s} }}{{\left( {\rho C_{p} } \right)_{f} }}} \right)} \right)$
Thermal conductivity	$\kappa_{nf} \, = \,\frac{{\kappa_{f} \left( {\left( {\kappa_{s} + 2\kappa_{f} } \right) - 2\phi \left( {\kappa_{f} - \kappa_{s} } \right)} \right)}}{{\left( {\left( {\kappa_{s} + 2\kappa_{f} } \right) + \phi \left( {\kappa_{f} - \kappa_{s} } \right)} \right)}}$

**Table 2:** Thermophysical properties of Ferrofluid ($Fe_{3} O_{4}$) with base fluid^50–52^.

Properties	$H_{2} O$/Water	$Fe_{3} O_{4}$ Ferrofluid
$\rho \;\;\left( {{\text{kg/m}}^{{3}} } \right)$	997.1	5180
$C_{p} \;\left( {{\text{JK}}^{ - 1} {\text{lkg}}} \right)$	4180	650
$\kappa \;\left( {{\text{Wm}}^{ - 1} /{\text{K}}} \right)$	0.6071	9.7

**Figure 2:**
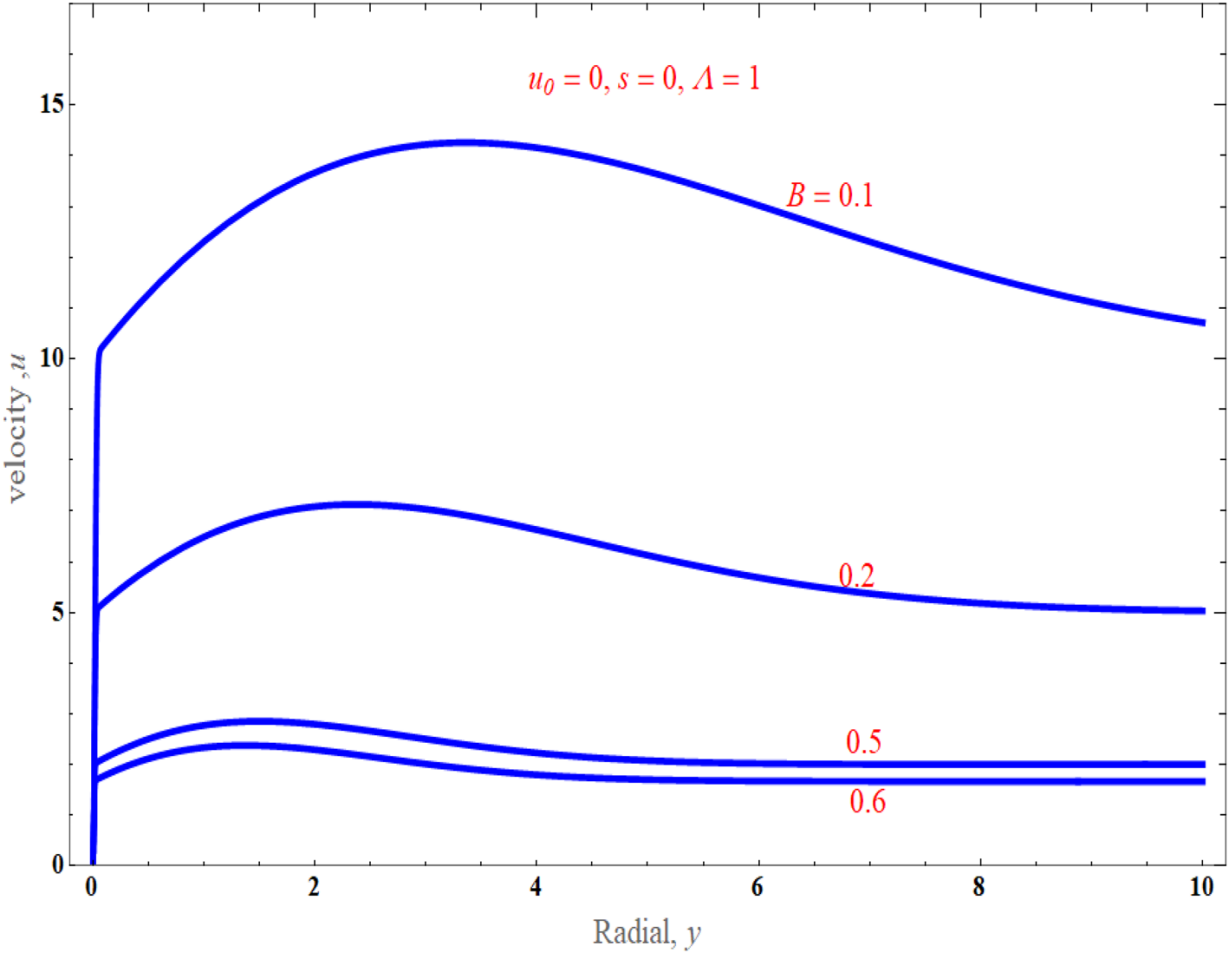
An impact of stretching parameter on normalized velocity distribution.

Figure 2 portrays how the stretching parameter $B$ affects the normalised velocity distribution (24). We noted that the stretching parameter enhances as velocity declines, keeping other parameter values at $u_{0} \, = \,0,\,s\, = \,0,\,\phi = 0.01,$ and $\Lambda \, = 1$. It is clear that $B$ has a dampening impact on the intensity of momentum, and the asymptotic behaviour at infinity is accurate. We also noticed that the momentum boundary layer declined with increased strength $B$.

**Figure 3:**
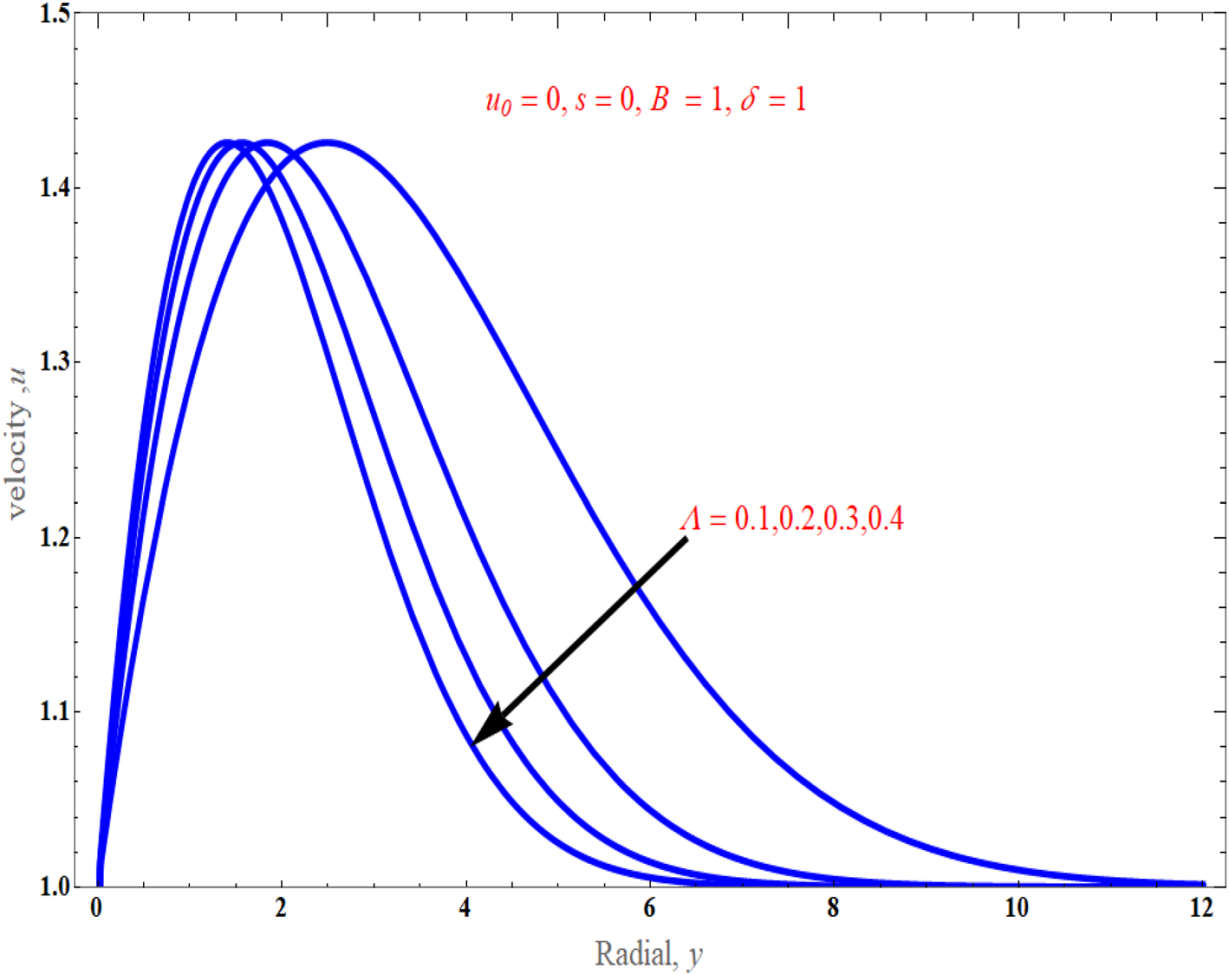
An impact of casson parameter on velocity distribution.

**Figure 4:**
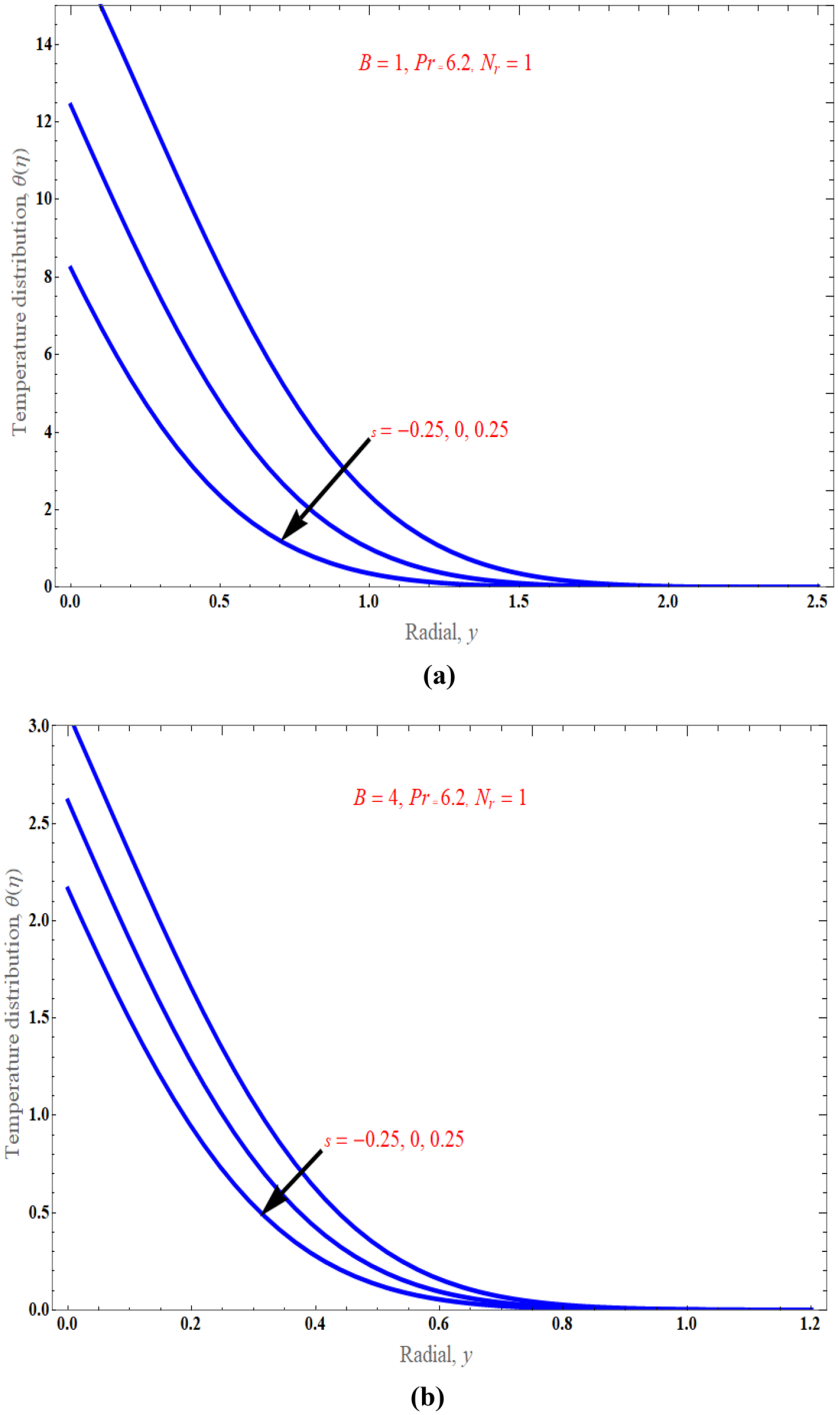
(**a**) An impact of suction/injection parameter on temperature distribution. (**b**) An impact of suction/injection parameter on temperature distribution.

**Figure 5:**
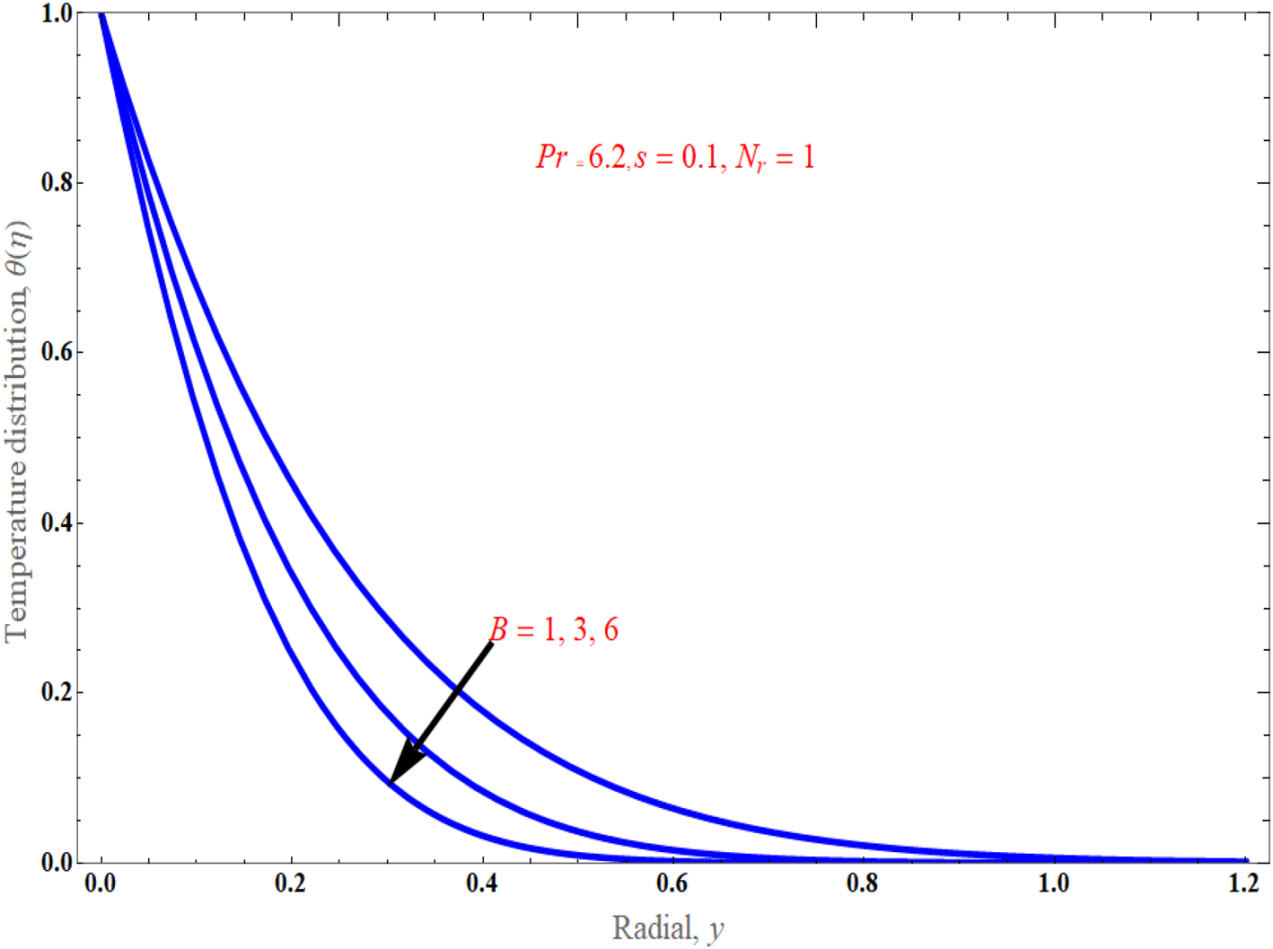
An impact of stretching parameter on temperature distribution.

Figure 3 depicts the influence of the casson parameter $\Lambda$ on the normal velocity profile. This figure revealed that as the strength of the casson parameter enhances, the nanoparticle flow diminishes gradually because the sheet stretchiness slows moving fluid components. Substantially, in this scenario, the momentum barrier layer thickness falls as $\Lambda$ grows. It is a known fact that increasing the Casson parameter decreases the yield stress of the Casson fluid, and increasing it indefinitely will make the fluid behave as a Newtonian fluid. It is evident that fluid motion is slowed down in velocity due to increase in the value of the Casson parameter $\Lambda$, which means decrease in the velocity profiles and leads to a decrease in the momentum boundary layer thickness. Figure 4a, b display the fluctuation in the temperature profile owing to the mass suction/injection parameter $s$, when $\Pr = 6.2,\,\phi = 0.01,\,$ and $N_{r} = 1$ with $B = 1$ and $B = 4$*.* the temperature is found to decrease as $s$ increases, and it decreases as the distance from the surface increases, and finally vanishes at some large distance from the surface. It is suggested that for case of suction $\left( {s > 0} \right)$, the increase of $s$ results to the boundary layer thinning while in the case of injection $\left( {s < 0} \right)$, the presence of injection effect has promoted to the drastically increases in the boundary layer thickness. As observed from Fig. 5, the thermal boundary layer drops when there is an increment in stretching parameter $B$ strength because stretching improves the nanofluid movement, which further raises the thickness of the thermal boundary layer, leading to significant thermal gradients on the surface.

**Figure 6:**
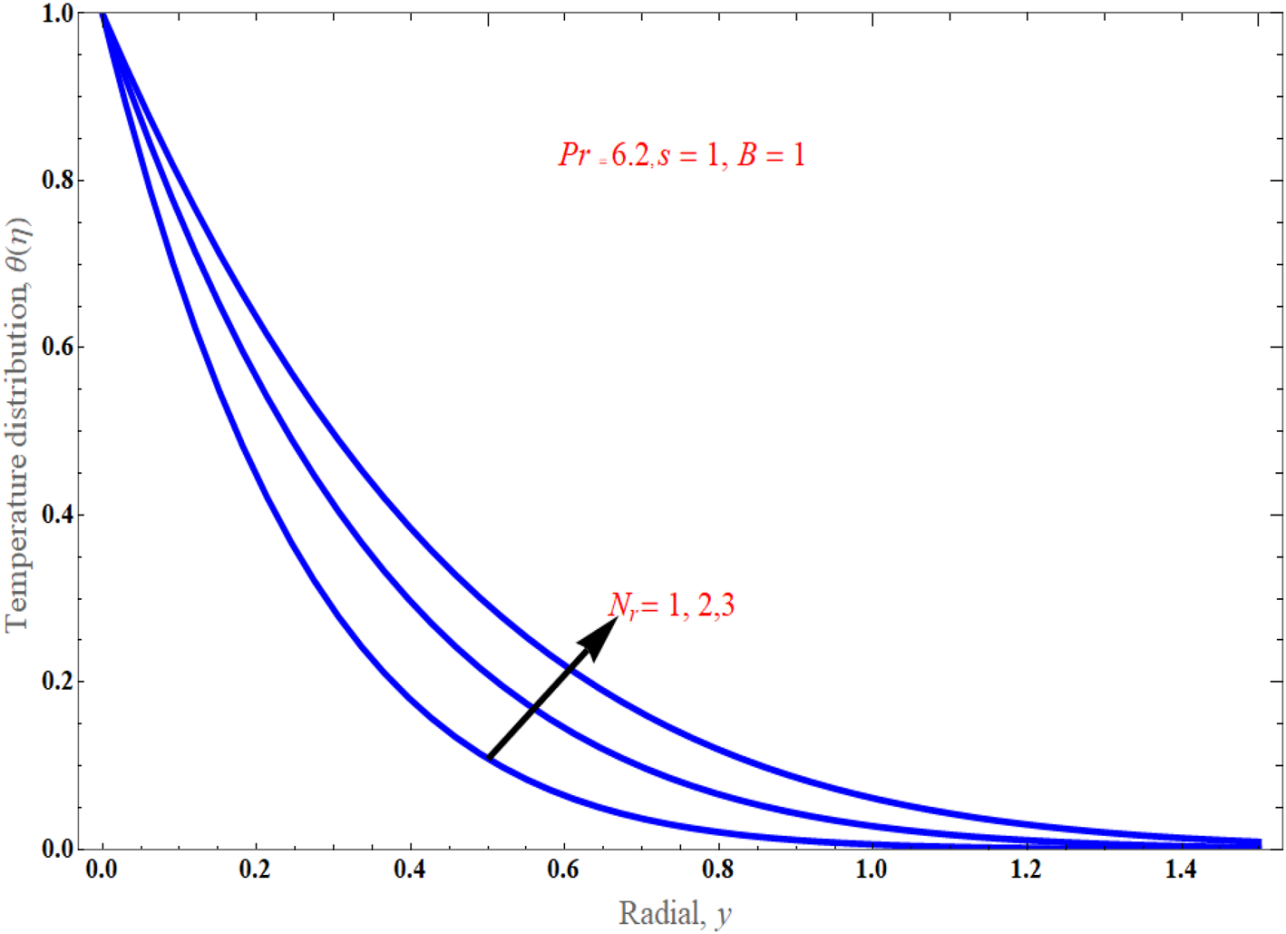
An impact of radiation on temperature distribution.

**Figure 7:**
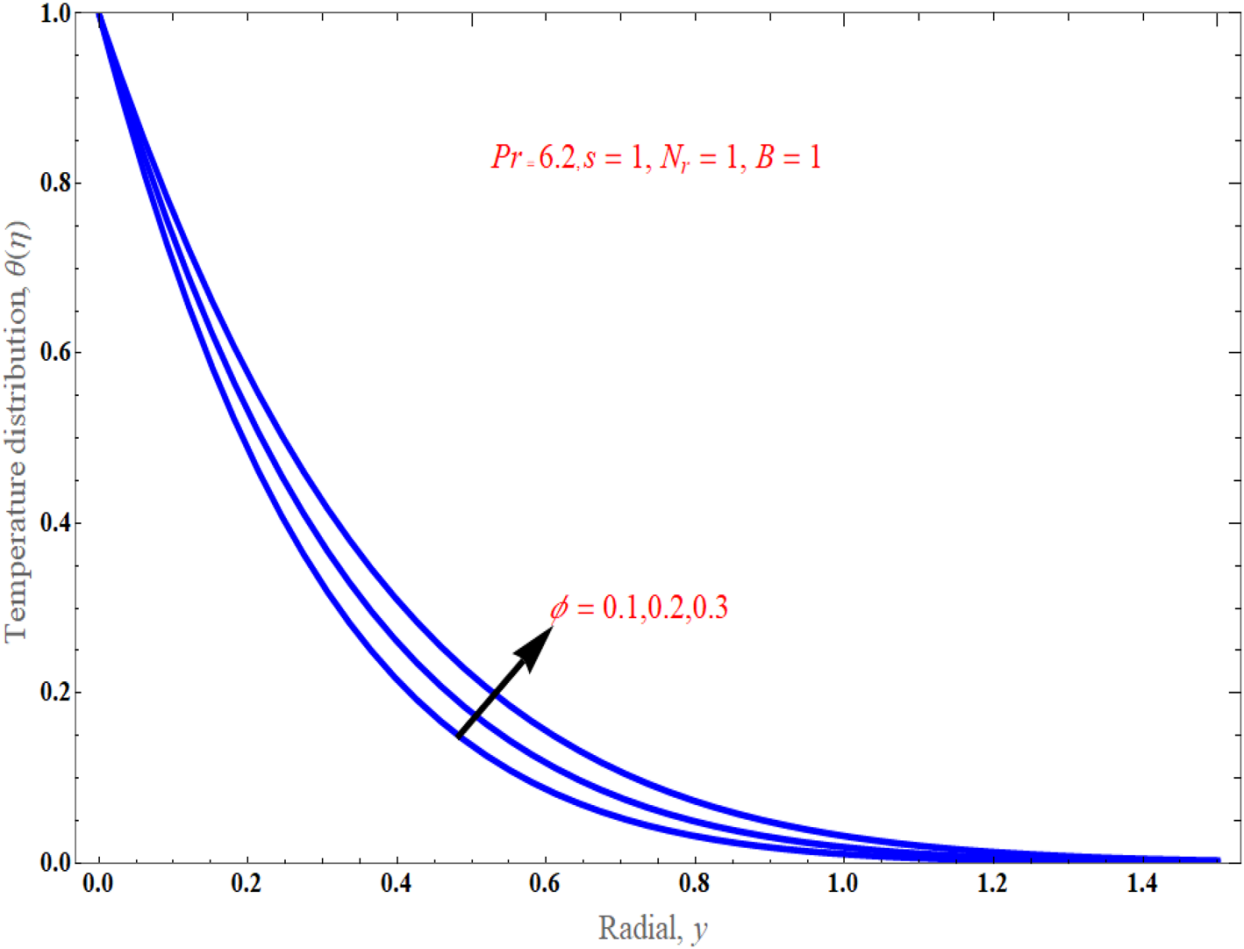
An impact of solid volume fraction on temperature distribution.

**Figure 8:**
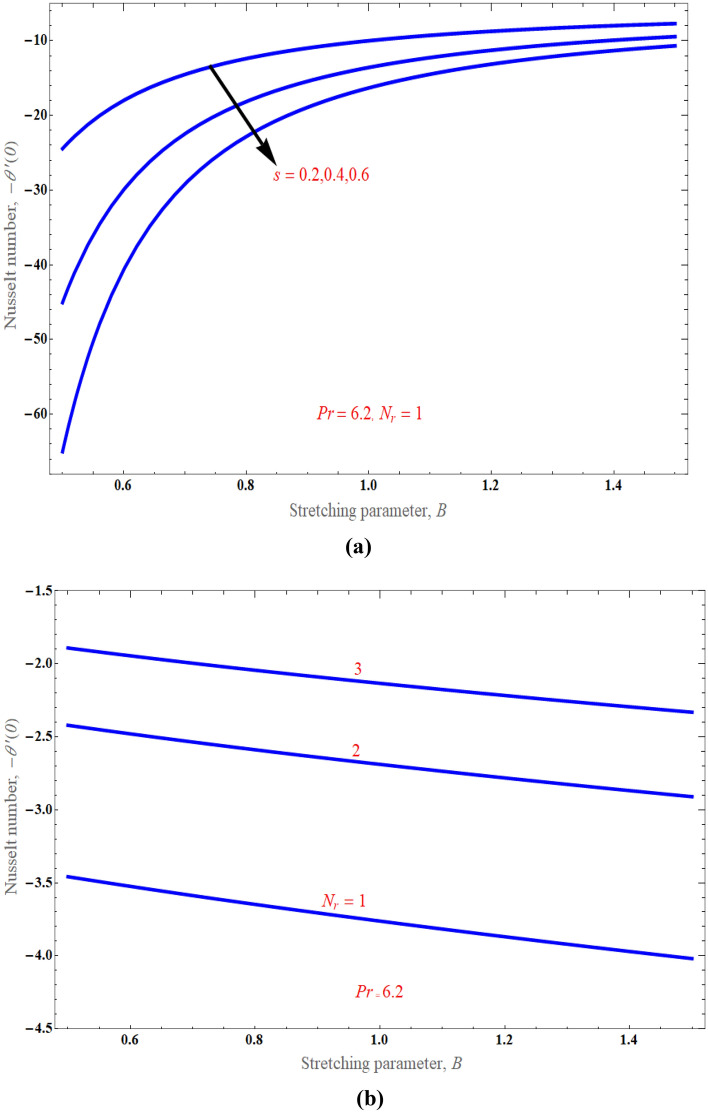
(**a**) An impact of suction/injection parameter on Nusselt number profile. (**b**) An impact of radiation on Nusselt number profile.

Figure 6, respectively, demonstrated the distribution of thermal radiation $N_{r}$ on temperature distribution. It has been noted that as the value of thermal radiation increases with temperature, so does the temperature distribution. Actually, radiation is a thermal transfer phenomenon that transmits heat via liquid particles. So it produces some heat in the flow. Hence, we perceive an increase in heat transfer for higher values of $N_{r}$. Figure 7 presents the consequences of solid volume fraction $\phi$ on temperature distribution. It is evident from figure, hence, it can be shown that by improvement in nanoparticle volume fraction with increasing the temperature boundary layer. It is obvious that whenever volume fraction of nanoparticle grows, the thermal conductivity and thermal diffusivity is also enhanced. Due to rising values of $\phi$, the temperature distributions become distorted resulting in an increase in the overall heat transfer. This result can be attributed to the dominance of the thermal conductivity property. It is worth noting that as the values of $\phi$ increase, the thickness of the thermal boundary layer near the top surface rises which indicates a steep temperature gradients. Figure 8a exemplify the characteristics of mass suction/injection parameter $s$ on reduced Nusselt number at some specific values of $\Pr = 6.2,\,\;\phi = 0.1$, and $N_{r} = 1$. Figure 8a display the rate of heat transmission declines uniformly on escalating the strength of the mass suction/injection. Rate of the heat transfer improved with incrementing value of radiation $N_{r} \,$ has been observed in Fig. 8b.

**Figure 9:**
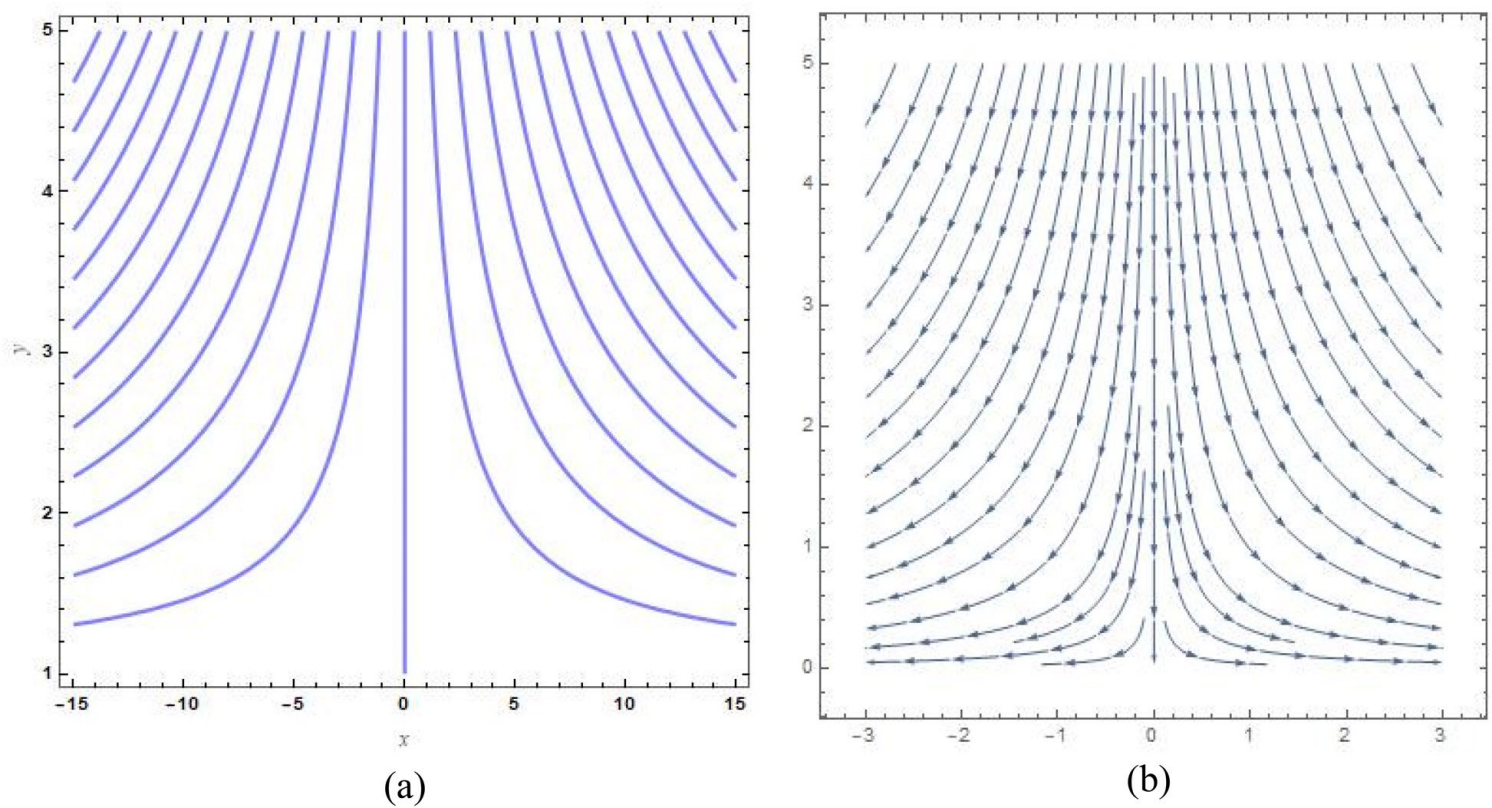
(**a**) Streamlines pattern and (**b**) vector field for $u_{0} \, = \,s\, = \,0,\,\Lambda \, = \,1\,$ and $B = 1$*.*

**Figure 10:**
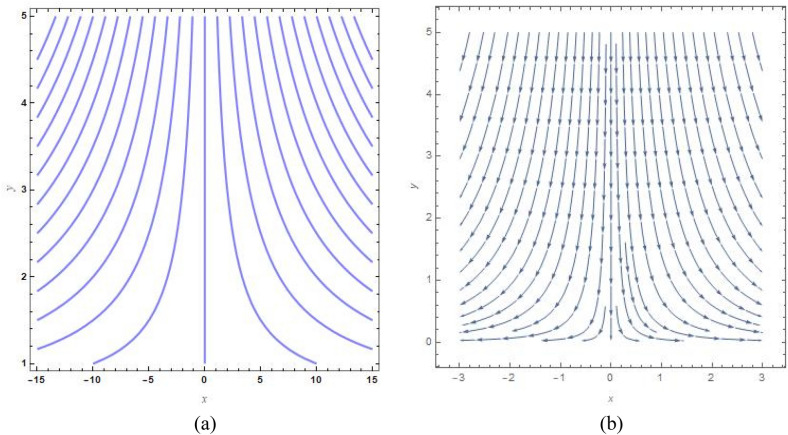
(**a**) streamlines pattern and (**b**) vector field for $u_{0} \, = \,s\, = \,0,\,\Lambda \, = \,1\,$ and $B = 2$*.*

**Figure 11:**
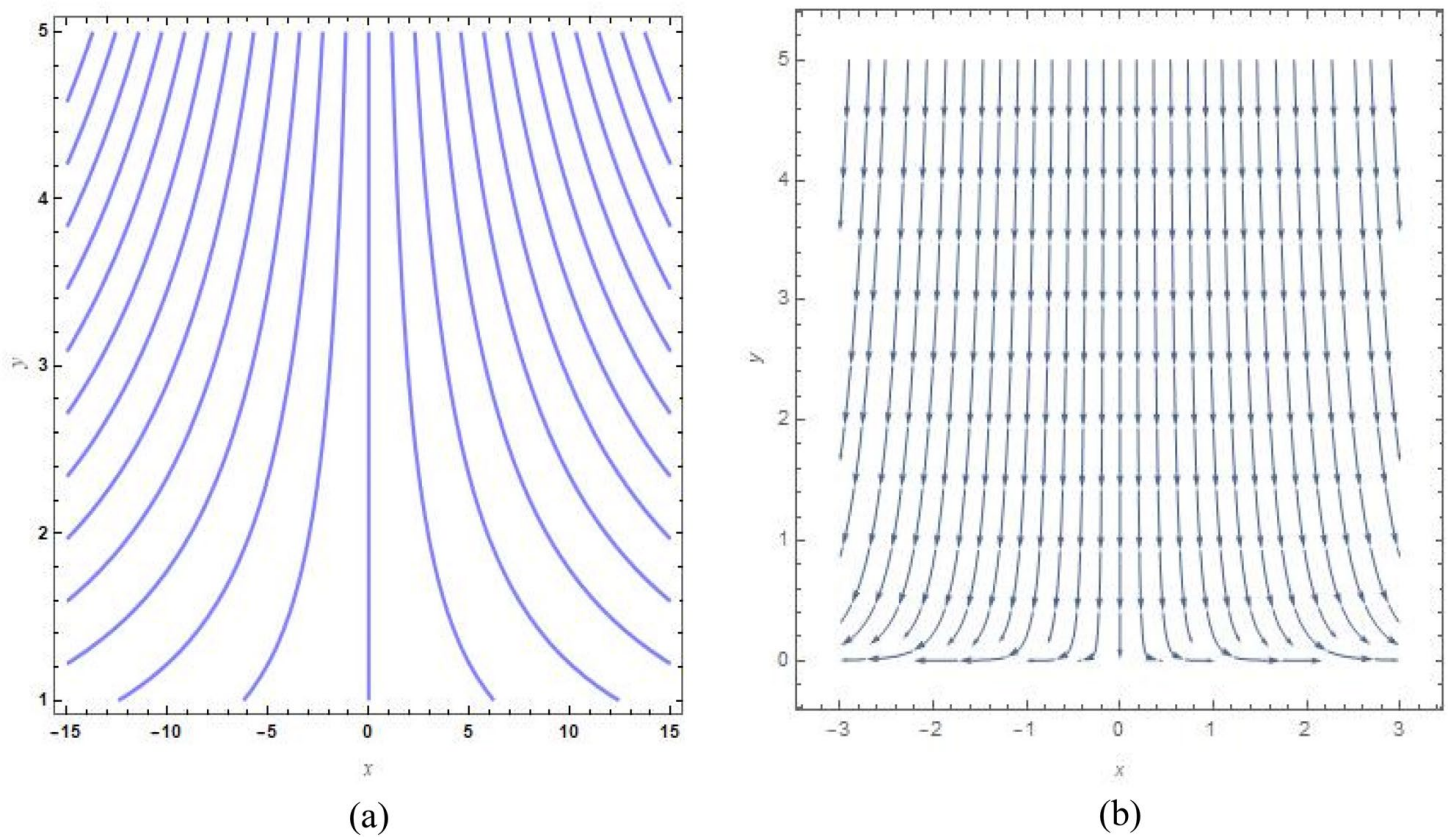
(**a**) Streamlines pattern and (**b**) vector field for $u_{0} \, = \,s\, = \,0,\,\Lambda \, = \,1\,$ and $B = 10$*.*

**Figure 12:**
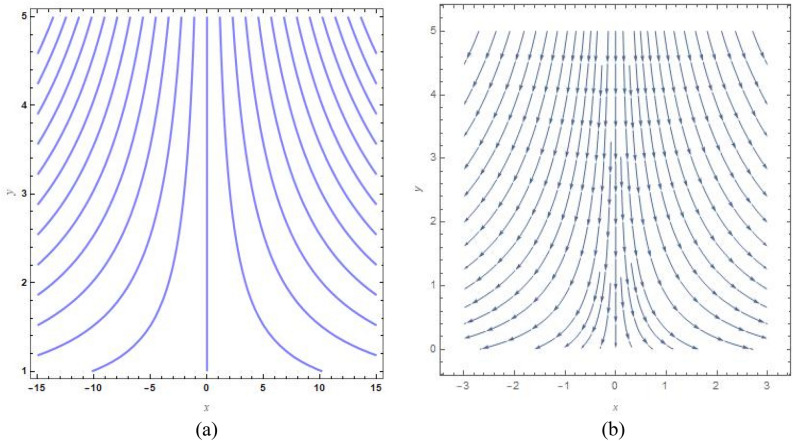
(**a**) Streamlines pattern and (**b**) vector field for $u_{0} \, = \,0,s\, = \,0.5,\,\Lambda \, = \,1\,$ and *B* = *1*.

**Figure 13:**
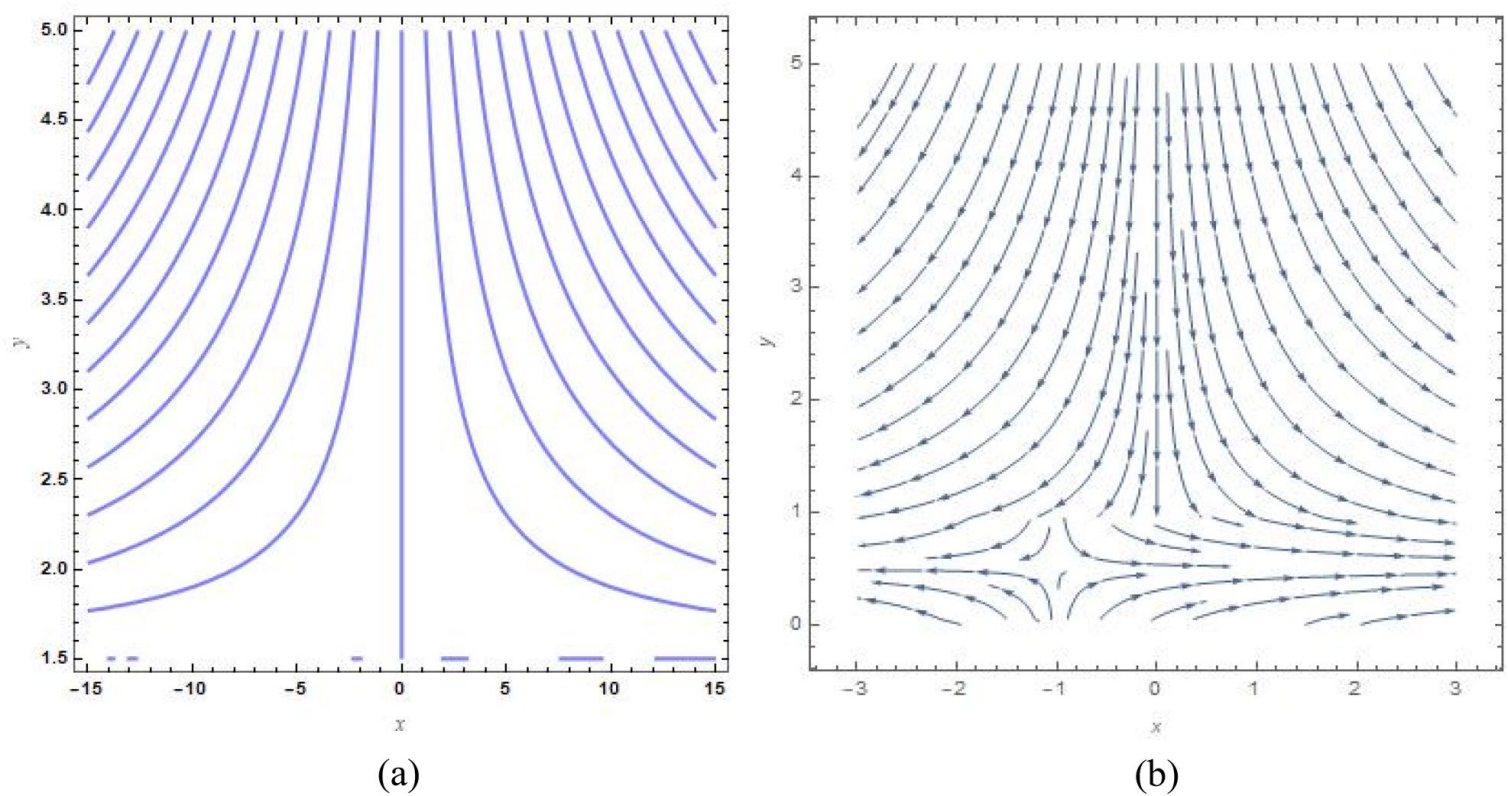
(**a**) Streamlines pattern and (**b**) vector field $u_{0} \, = \,0,s\, = \, - 0.5,\,\Lambda \, = \,1\,$ and *B* = *1.*

**Figure 14:**
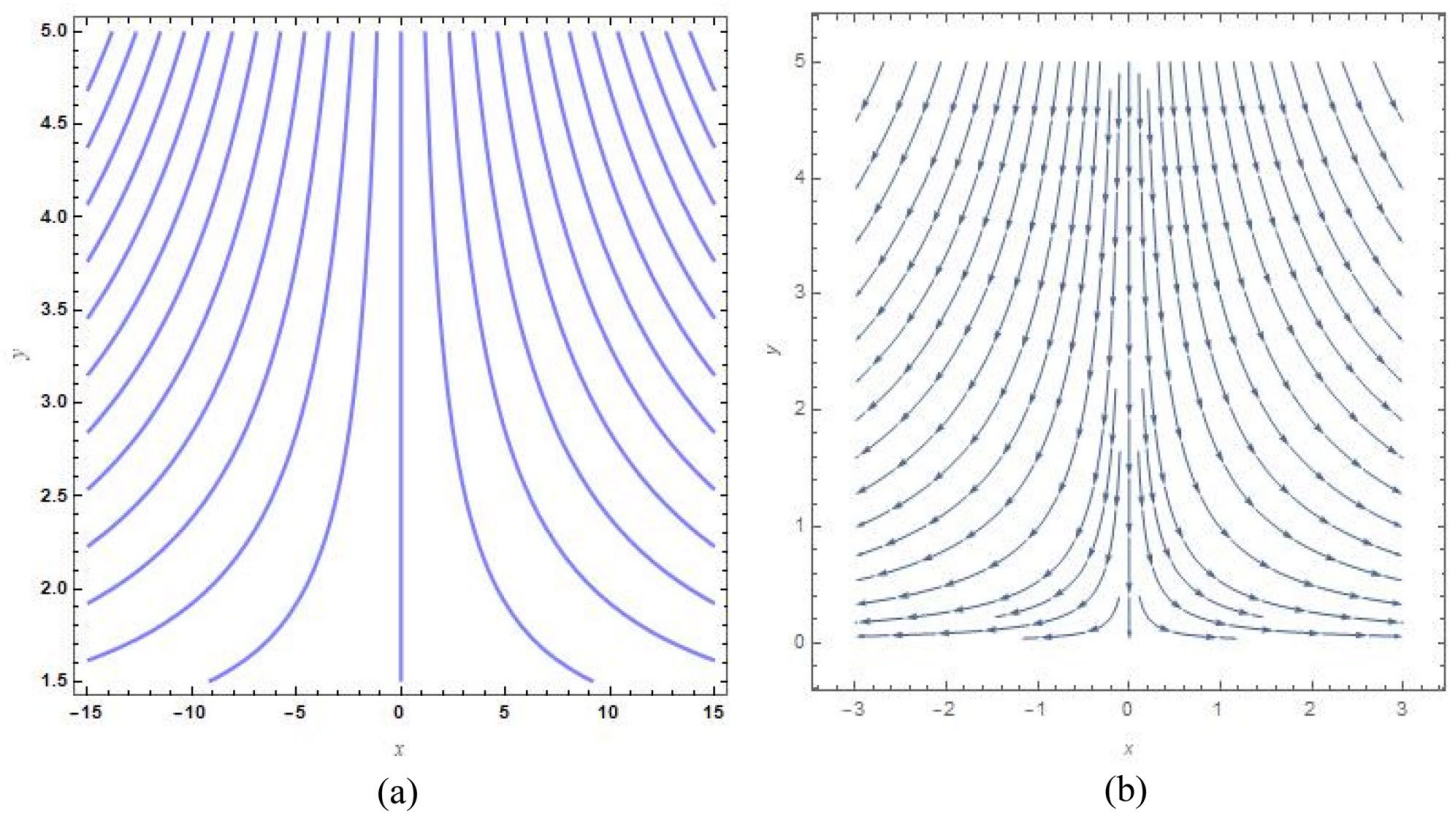
(**a**) Streamlines pattern and (**b**) vector field for $u_{0} \, = \,0,$ and $B = 1$.

**Figure 15:**
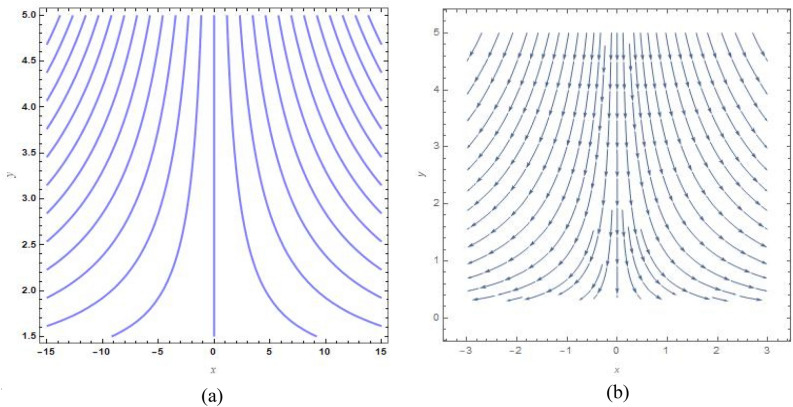
(**a**) Streamlines pattern and (**b**) vector field for $u_{0} \, = \,1,$ and $B = 1$.

Figure 9a, b show the analogous streamlines and vector field if $u_{0} \, = \,s\, = \,0,\,\Lambda \, = \,1\,$ and $B = 1$. When the axial velocities at the flat plane as well as far above are different, we notice that an oblique stagnation happens. Figures 10a, b and 11a, b shows the impact of increasing $B$ values on the streamlines and velocity fields, respectively with $u_{0} \, = \,s\, = \,0,\,\Lambda \, = \,1\,$ with $B = 2$ and $B = 10$. As anticipated by Fig. 2, The development of a orthogonal stagnation-point flow results from the rapid reduction of $U$ under the influence of the enormous value of *B*. From above Figures represent the provoking behaviour of stretching parameter through streamlines and vector field. It has been seen that upsurging data of stretching parameter $B$, helps to accelerate the motion of the fluid. Stretching escalates the motions of the liquid particles. Similar behaviours are observed in the following Figs. 12a, b, 13a, b, 14a, b and 15a, b.

The streamlines and vector field for a wall suction at $s\, = \,0.5$ are presented in the Fig. 12a, b with $B = 1$. the results in Fig. 13a, b are produced by wall injection at $s\, = \, - 0.5$ with $B = 1$. In accordance with the actual prediction, wall suction regularises fluid flows and encroachment takes place at certain downstream points. Nevertheless, injection promotes streamline divergence at the radial position $y\, = \, - s$. Assuming $B = 0$, we predict that the thermal solution for the stretched plate is determined by

33 $$\theta \left( y \right)\, = \,e^{{ - \frac{{\Pr sA_{2} y}}{{\left( {A_{1} + N_{r} } \right)}}}} ,$$

and the accompanying Nusselt number is denoted as

34 $$Nu_{x} \, = \,\frac{{\Pr sA_{2} }}{{\left( {A_{1} + N_{r} } \right)}},$$

regarding the stretched cylinder, Fig. 14a, b show the streamlines with normalized velocity field for $u_{0} \, = \,0$ and $B\, = \,1$ along with Fig. 15a, b characterized the nanofluid flow pattern and normalized vector field for $u_{0} \, = \,1$ and $B\, = \,1$. A quite different character of flow field is anticipated as compared to the stretching plate, refer to Fig. 9a, b.

In relation to the stretching flat surface**,** initially, we observe that in the scenario of $B\, = \,0$ in (23), It is conceivable to put $\delta \, = \,0$, and as a result, we arrive at the following efficient solution for the Navier’s-Stokes equations (2–5)

35 $$u\, = \,u_{0} e^{{ - \frac{s\Lambda }{{\delta \left( {1 + \Lambda } \right)}}}} ,\;\;v\, = \, - s,\;\;p\, = \,p_{0} ,$$

**Figure 16:**
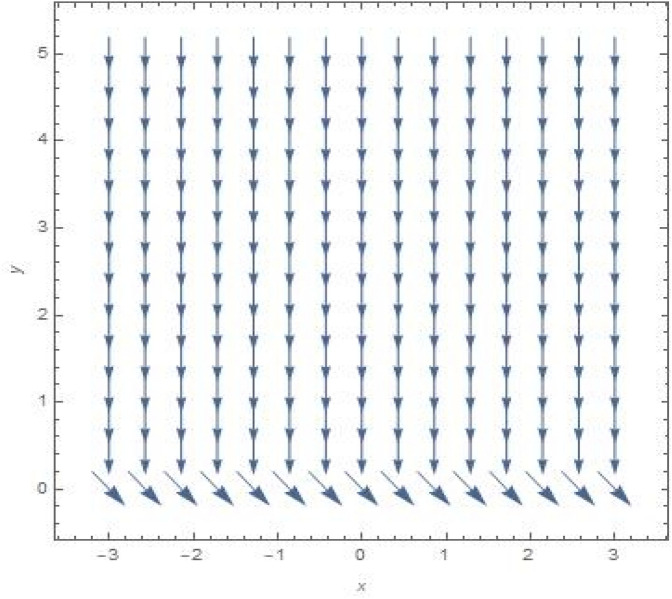
Suction velocity field with asymptotic behaviour for $u_{0} \, = \,s\, = \,1$,if $B = \,\delta \, = \,0$.

It is commonly referred as the asymptotic suction solution $\left( {s > 0} \right)$, for the uniform far-field stream. In this case no stagnation occurs and the velocity field is shown in Fig. 16.

## Concluding remarks

The current study discusses an impact of thermal radiation on stagnation point flow of non-Newtonian Casson nanofluid ($Fe_{3} O_{4}$) through a flat stretching plate/circular cylinder with mass suction/injection. The momentum and energy equations are written as system of ODEs employing Eqs. (10, 11) and then solved analytically. Additionally, the walls are exposed to injection or suction as indicated by the analytical solutions; in the absence of stretching, these solutions trend to the well-known specific limits. The exact solution for the temperature and Nusselt number are also determined. Plots are employed to discuss the influence of appropriate parameters on the described flow. The key observations of this work are shortly written as:

- Because the surfaces are extending, axial velocities are reduced.
- In this work both fluid flow and pressure are dependent on generalized coordinates.
- In accordance with physical characteristics, the stagnation-point phenomenon is either oblique or orthogonal.
- Wall suction is leads to diminishes in the temperature distribution as compared to the wall injection.
- The flow velocity becomes diminishes by strengthening the Casson fluid parameter $\Lambda$ and stretching parameter $B$.
- When $\Lambda$ tends to infinity, it reduce to the results of Newtonian case.
- It is observed that solid volume fraction $\phi$ and radiations $N_{r}$ increase with increased the thermal boundary layer thickness, while stretching parameter $B$ and mass suction/injection $s$ increase with decreased the thermal boundary layer thickness.

Several earlier works form a limiting case of the present study:

- $$\mathop {\lim }\limits_{\begin{subarray}{l} \Lambda \to \infty , \\ \phi \, \to 0, \\ q_{r} \to 0, \end{subarray} }$${Results of present works}$\to${Results of Turkyilmazoglu^8^}.
- $\mathop {\lim }\limits_{\Lambda \to \infty ,}${Results of present work}$\to${Results of Parvin^52^}.

Further extensions of the current work can be implemented incorporating new physical mechanisms, such as an external magnetic field, porous media, couple stress fluid or non-Newtonian fluid rheology.

## Data Availability

Data that support the findings of this study are available from the corresponding author upon reasonable request.

## List of symbols

$$A_{1} ,\,A_{2}$$: Constants (-)
$$B$$: Stretching parameter (-)
$k^{*}$: Mean absorption coefficient (-)
$$N_{r}$$: Radiation (-)
$$p$$: Pressure $$\left( {N/m^{2} } \right)$$
$$\Pr$$: Prandtl number (-)
$q_{r}$: Radiative heat flux $\left( {W/m^{2} } \right)$
$$r$$: Radial axis (-)
$$R$$: Pipe radius $$\left( m \right)$$
$$s$$: Permeability $$\left( {N/A^{2} } \right)$$
$$T$$: Fluid temperature $$\left( K \right)$$
$$T_{w}$$: Wall temperature $$\left( K \right)$$
$$T_{\infty }$$: Ambient temperature $$\left( K \right)$$
$$\left( {u,v} \right)$$: Velocity components $$\left( {m/s} \right)$$
$$u_{0}$$: Constant (-)
$$v_{0}$$: Constant (-)
$$\left( {x,y} \right)$$: Coordinate system (-)

## Greek symbols

$\Lambda$: Casson fluid parameter (-)
$$\alpha$$: Constant (-)
$$\delta_{1}$$: Constant (-)
$$\delta$$: Pressure parameter $$\left( {N/m^{2} } \right)$$
$\rho_{nf}$: Density of nanofluid $\left( {Kg/m^{3} } \right)$
$\kappa_{nf}$: Thermal conductivity of nanofluid $\left( {Wm^{ - 1} K^{ - 1} } \right)$
$$\mu_{nf}$$: Dynamic viscosity $$\left( {Ns/m^{2} } \right)$$
$\left( {\rho C_{p} } \right)_{nf}$: Heat capacitance of nanofluid $\left( {J/K} \right)$
$$\nu_{nf}$$: Kinematic viscosity $$\left( {m^{2} /s} \right)$$
$\phi$: Solid volume fraction (-)
$$\Gamma$$: Dummy variable (-)

## Subscripts

$$f$$: Base fluid (-)
$$nf$$: Nanofluid (-)
$$CNTs$$: Carbon nanotubes (-)

## Abbreviations

MHD: Magnetohydrodynamic (-)
ODEs: Ordinary differential equations (-)
PDEs: Partial differential equations (-)
SPF: Stagnation point flow (-)
BLF: Boundary layer flow (-)
NSEs: Navier–Stokes equations (-)
